# Positive Organizational Psychology in Nursing Education: A Bibliometric Analysis and Narrative Review (2005–2025)

**DOI:** 10.1155/jonm/9914092

**Published:** 2026-10-17

**Authors:** Wanqi Ou, Shuibing Cai, Ming Chen, Qiaoyan Guan, Haiyi Liang, Ying Cao, Yueya Wang, Haozhang Huang, Xuting Chen

**Affiliations:** ^1^ Department of Nursing Guangdong Mental Health Center Guangdong Provincial People’s Hospital (Guangdong Academy of Medical Sciences) Southern Medical University Guangzhou 510120 China fimmu.com; ^2^ Department of Psychiatry Guangdong Mental Health Center Guangdong Provincial People’s Hospital (Guangdong Academy of Medical Sciences) Southern Medical University Guangzhou 510120 China fimmu.com; ^3^ Guangdong Provincial Geriatrics Institute Guangdong Provincial People’s Hospital (Guangdong Academy of Medical Sciences) Southern Medical University Guangzhou 510080 China fimmu.com

**Keywords:** bibliometric analysis, narrative review, nursing education, nursing students, positive organizational psychology, psychological capital, resilience

## Abstract

**Background:**

Positive organizational psychology (POP) offers a strengths‐based framework for fostering psychological resources, well‐being, engagement, and supportive organizational climates—increasingly relevant to nursing education, where students and educators face persistent academic, emotional, and workplace pressures. The global development and knowledge structure of POP‐related nursing‐education research remain unmapped.

**Methods:**

We conducted a bibliometric analysis combined with a narrative synthesis. The Web of Science Core Collection was searched on 6 August 2026 (2005–2025) using a three‐tier strategy validated against a frozen seed set (verified recall 96%) and selected by a prespecified precision duel. Screening followed PRISMA 2020 with independent double‐blind review and an exclusion audit; 2321 of 13,658 records were included. Analyses used bibliometrix; a narrative synthesis interpreted the resulting themes.

**Results:**

Publications grew from 4 in 2005 to 495 in 2025 (compound annual growth 27.2%), accelerating after 2020. China (472) and the United States (444) were the largest contributors, and China accumulated the most citations (9940). Nurse Education Today, BMC Nursing, and Nurse Education in Practice were the most productive journals. After deterministic author‐name disambiguation, Tracy Levett‐Jones was the most productive author (14 publications). Eight thematic communities were identified, centered on simulation‐based education and self‐efficacy, stress and mental health, resilience and coping, emotional intelligence, the clinical learning environment, learning approaches, well‐being, and COVID‐19/professional identity; professional identity and online learning were the strongest recent trend topics.

**Conclusions:**

POP‐related nursing‐education research has expanded rapidly, particularly around stress, resilience, self‐efficacy, and professional identity. Future work should move beyond descriptive designs toward theory‐driven, longitudinal, intervention, and cross‐cultural studies.

**Implications for Nursing Management:**

Nurse managers and academic leaders can use this map to embed positive organizational resources—psychological capital, supportive climates, and positive leadership—into curricula, faculty development, and clinical learning environments, with priority on strengthening professional identity and well‐being.

## 1. Introduction

Nursing education is central to the preparation of a competent, resilient, and sustainable nursing workforce [1, 2]. Nursing programs operate in increasingly complex academic and clinical environments: students integrate theoretical knowledge, technical skills, and ethical reasoning while adapting to demanding clinical placements [3], and educators face curriculum reform, digital transformation, and growing student‐support demands [4, 5]. These pressures contribute to stress, burnout, reduced engagement, and weakened professional identity [6].

Positive psychology studies optimal human functioning, shifting attention from pathology and dysfunction to strengths, well‐being, and flourishing [7, 8]. Positive organizational psychology (POP) extends this orientation to organizational settings, examining how individuals, teams, and institutions function optimally through positive resources and supportive climates [9]. POP‐related constructs include psychological capital, resilience, self‐efficacy, work engagement, psychological safety, positive leadership, and organizational support [10–12].

Applying POP to nursing education is theoretically and practically important: it may strengthen students’ confidence, coping, professional identity, and readiness for clinical learning; promote educators’ engagement, well‐being, and retention [13, 14]; and highlight supportive learning climates, psychologically safe clinical education, mentoring, and institutional practices that enable both groups to flourish [15, 16]. POP thus offers a framework for addressing persistent challenges in nursing education without framing students and educators solely through deficit perspectives.

Existing studies have examined many of these topics in nursing and health‐professions education, yet the literature remains conceptually dispersed: most studies address isolated constructs or specific educational contexts, and few have systematically mapped the global landscape and thematic evolution of POP‐related research in nursing education over time.

Therefore, this study aimed to map global research trends and emerging themes in POP‐related nursing education research from 2005 to 2025 using bibliometric analysis and narrative synthesis. Specifically, it sought to (1) describe publication growth and citation patterns; (2) identify leading countries, institutions, authors, and journals; (3) examine collaboration networks and keyword co‐occurrence; (4) characterize thematic evolution; and (5) synthesize established and emerging themes to inform future research and practice.

## 2. Methods

### 2.1. Study Design and Reporting

This study combined a bibliometric analysis with a narrative synthesis. The bibliometric component was used to map the scientific production, collaboration structure, citation impact, and keyword evolution of research on POP in nursing education; the narrative component was used to interpret the major empirical and conceptual themes identified from the bibliometric results. Reporting follows the PRISMA 2020 statement [17], the PRISMA‐S extension for literature‐search reporting [18], and the BIBLIO guideline for bibliometric reviews of the biomedical literature [19]; the completed BIBLIO checklist is provided in Table S1. The eligibility criteria, screening rules, tiered search‐validation plan, and analytic parameters were fixed in a prespecified protocol before screening began, and every subsequent amendment was logged with a time‐stamped audit trail. Because this study used published bibliographic records and did not involve human participants or individual‐level data, ethical approval was not required.

### 2.2. Data Source

Publications were retrieved from the Web of Science Core Collection (WoSCC), which was selected because of its broad multidisciplinary coverage, standardized citation metadata, and compatibility with bibliometric software. A single database was used to ensure consistency in bibliographic fields and to reduce potential duplication and format discrepancies across databases [20]. The search covered the editions subscribed to by the authors’ institution (Science Citation Index Expanded, Social Sciences Citation Index, Arts and Humanities Citation Index, and Emerging Sources Citation Index) and was executed on 6 August 2026, covering publication years from 1 January 2005 to 31 December 2025. No language restriction was applied at the search stage; language eligibility was assessed during screening.

### 2.3. Search Strategy: A Validated Three‐Tier Design

Because POP is a multiconstruct umbrella field without a controlled vocabulary, we designed and validated a three‐tier search in which the nursing‐education context facet was held constant across tiers while the POP construct facet varied in field restriction and vocabulary width so that differences in recall and precision could be attributed solely to the construct facet (Tables S2‐S3). The construct vocabulary was derived empirically from the full texts of a frozen seed set of 50 eligible publications and the indexing terms of six peer‐reviewed source reviews, not stipulated a priori. The seed set (assembled before any search testing; 15 engagement/motivation, 14 mindfulness, 13 resilience, 7 well‐being/self‐care, and 1 professional‐identity publications, stratified across 2005–2014/2015–2019/2020–2025) was verified record‐by‐record against WoSCC, with one prespecified replacement for a journal not yet indexed. No NOT operators were used in any tier, so that all exclusions remained auditable at the screening stage. Tier 1 (strict) restricted construct terms to the title field. Tier 2 (moderate) searched the same construct vocabulary—plus second‐tier positive‐psychology constructs (positive emotion∗, positive affect, sense of coherence, belonging∗, self‐esteem, self‐determination, intrinsic motivation, personal growth, meaning in life, gratitude, compassion satisfaction, and academic satisfaction)—in the topic field (title, abstract, author keywords, and Keywords Plus). Tier 3 (broad) additionally included high‐noise terms (cope, coping, stress∗, burnout∗, gamif∗, serious game∗, escape room, academic success, retention, and persist∗) as a recall ceiling.

Tier selection followed a prespecified decision rule: a tier qualified if it achieved ≥ 90% recall against the frozen seed set, and among qualifying tiers, the one with the highest precision in a stratified random sample (*n* = 300 per tier; 100 records per period 2005–2014, 2015–2019, and 2020–2025) was selected. Verified seed recall was 62% (31/50) for Tier 1—below threshold, because engagement/motivation studies typically name only the intervention format (e.g., serious games, virtual reality, and simulations)—96% (48/50) for Tier 2, and 98% (49/50) for Tier 3 (Tables S4). In the precision duel, two independent screeners double‐coded all 600 sampled records; after codebook calibration and principal‐investigator arbitration of 42 residual disagreements, agreement was 93.0% and Cohen’s *κ* was 0.811. Final precision was 26.3% (95% CI 21.7%–31.6%) for Tier 2 versus 18.3% (95% CI 14.4%–23.1%) for Tier 3, and the decision was robust to worst‐case arbitration scenarios (Tier 2 31.7% vs. Tier 3 21.7%) (Table S5). Tier 2 was therefore locked as the final search strategy. The complete search strings for all three tiers are provided in Table S2.

### 2.4. Eligibility Criteria, Screening, and Selection Audit

Publications were eligible if they (1) were published between 2005 and 2025; (2) were journal articles or reviews; (3) were written in English; (4) addressed a nursing‐education context (nursing students, nurse educators, or formal nursing programs; exclusion code E1 otherwise); and (5) showed substantive focus on at least one POP construct as a measured outcome, as the content of an educational intervention, or as a primary qualitative theme (exclusion code E2 otherwise). Records addressing mixed populations in which nursing participants could not be separated were excluded (E3), as were ineligible document types (E4) and non‐English records (E5); the full codebook (with all amendments) is provided in Table S6.

Records were processed in three stages. First, removing 13 duplicate records and prefiltering removed 1284 records: 311 non‐English, 372 non‐research document types, 366 conference proceedings or book chapters, 15 retracted publications, and 220 early‐access records that had been formally published in 2026 and therefore fell outside the publication‐year window. Second, the remaining records underwent independent double‐blind title/abstract screening by two screeners (Shuibing Cai and Haozhang Huang) under the piloted codebook: a 500‐record pilot batch calibrated the codebook (125 disagreements resolved; six records referred to the principal investigator [Xuting Chen]), followed by 13 full‐scale batches. Observed agreement across batches ranged from 0.76 to 0.84. Cohen’s *κ* varied across batches (0.11–0.63) because the safety‐net screener’s inclusion‐side false‐positive rate increased across batches (from 40% to 82%) as the corpus progressed into constructs with lower base rates—an asymmetry known to depress *κ*; all disagreements were therefore adjudicated record by record against the codebook rather than resolved statistically, and residual conflicts were decided by the principal investigator. Third, an independent audit of a random sample of 200 excluded records (196 within the window) rescued three records, yielding a false‐exclusion rate of 1.5%, below the prespecified 2% threshold (as shown in Table S7).

Following PRISMA 2020 (Figure S1), 13,658 records were identified; after deduplication (*n* = 13), prefiltering (*n* = 1284), and title/abstract screening of 12,361 records (10,029 excluded), 2332 records advanced to the eligibility stage. Of these, 16 records of uncertain eligibility were assessed at full text; 11 were excluded (nine for insufficient POP‐construct evidence, two for population mismatch), including four records re‐assessed by the principal investigator at full‐text level during revision. The final dataset therefore comprised 2321 publications.

### 2.5. Bibliometric Analysis

Bibliometric analyses were performed with the R package bibliometrix Version 4.3.0 in R Version 4.5.1 [21]. The biblioAnalysis() and summary() functions were used to describe annual publication output, growth, document characteristics, citation patterns, and productive journals, authors, institutions, and countries. Country‐level analyses used the corresponding author’s country (single‐country vs. multiple‐country publications, and SCP/MCP), and journal productivity and impact were examined using publication counts, Bradford’s law, and collection‐based h‐indices (i.e., h‐indices computed within the present dataset and not career h‐indices).

Author names were disambiguated before any author‐level analysis using a four‐step deterministic procedure: (1) full‐name parsing with diacritic folding and surname anchoring (identical full names were treated as the same person); (2) hard merging of name variants sharing an ORCID; (3) affiliation‐anchored absorption of variants with the same surname, compatible given‐name initials, and either overlapping affiliations or co‐author bridges (144 variant pairs in 137 groups, each with documented evidence); and (4) a hard block preventing any merge when both records carried different ORCIDs. Residual collision risk was bounded at ≤ 21 name keys (0.27% of 7728 disambiguated authors), all in the long tail; no author in the top 20 was affected (as shown in Table S8). Institutional productivity was counted at the document level using WoS Organizations‐Enhanced names (each institution counted at most once per document).

Keyword analyses combined author keywords (DE) and Keywords Plus (ID) after normalization (lower‐casing, punctuation and whitespace harmonization, and synonym mapping). Keyword co‐occurrence networks were clustered with the Louvain algorithm [22]. For thematic evolution, the study window was divided a priori into three descriptive periods (2005–2013, 2014–2020, 2021–2025) on the basis of inflection points in the publication curve and the external COVID‐19 context; these periods are descriptive and were not derived from, or subjected to, formal change‐point inference. As a sensitivity analysis, a data‐driven two‐breakpoint segmented regression of log‐transformed annual output identified optimal breakpoints at 2012 and 2017, and thematic evolution was re‐run under the alternative segmentation (2005–2012, 2013–2017, and 2018–2025): of the 21 a priori thematic anchor keywords, the sets assigned to corresponding periods overlapped by 11, 17, and 19, respectively, and the five core thematic domains (simulation‐based teaching and self‐efficacy; stress and mental health; resilience and coping; emotional intelligence and empathy; professional identity) and the emergence order of newer topics (online learning, virtual reality, and COVID‐19) were unchanged, indicating that the thematic narrative is robust to the choice of period cut‐points.

### 2.6. Narrative Synthesis

The narrative synthesis followed the four‐element framework [23]. (1) Theory development: POP constructs were mapped onto positive‐psychology and organizational‐behavior theory before interpretation. (2) Preliminary synthesis: the keyword communities, thematic‐evolution clusters, trend topics, and the most cited publications were tabulated and organized into candidate themes. (3) Exploration of relationships: each candidate theme was examined against study designs, populations, and educational settings in the underlying publications to distinguish established themes (recurrent, measurement‐based constructs such as stress, resilience, psychological capital, engagement, and well‐being) from emerging themes (recently accelerating topics such as professional identity, online and simulation‐based pedagogies, and virtual reality). (4) Assessment of robustness: themes that rested on bibliometric signals alone without a coherent body of eligible publications were excluded from the synthesis.

## 3. Results

### 3.1. General Characteristics and Publication Growth

A total of 2321 publications met the eligibility criteria and were included in the bibliometric analysis (Figure S1). The dataset spans 2005–2025 and covers 377 journals from 83 countries, with 93,672 cited references. The included publications accumulated 43,585 citations (mean 18.78 per document; mean document age 4.5 years), and 17.0% involved international co‐authorship (395 multiple‐country vs. 1896 single‐country publications; affiliation country was not machine‐parsable for the remaining 30 records, 1.3%). Annual publication output grew from four publications in 2005 to 495 in 2025, with a compound annual growth rate of 27.2% (Figure 1). Output remained low and fluctuating through 2013 (≤ 35 publications per year), increased steadily from 2014 (47) to 2019 (92), and accelerated markedly after 2020, more than doubling from 123 publications in 2020 to 280 in 2022 and peaking at 495 in 2025.

**FIGURE 1:**
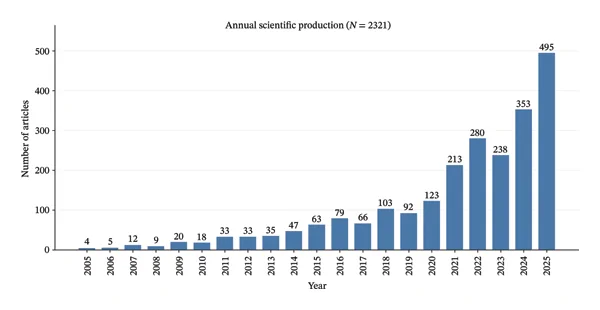
Annual number of publications on positive organizational psychology‐related research in nursing education, 2005–2025 (*N* = 2321).

### 3.2. Global Trends and Country Collaboration

The geographical distribution showed broad international engagement but uneven productivity (Figure 2A). China was the most productive country (472 publications), followed by the United States (444), Turkey (243), South Korea (181), and Australia (161); Saudi Arabia (125), Spain (122), Iran (113), the United Kingdom (110), and Canada (89) completed the top 10. Temporal analysis (Figure 2B) showed that China’s output rose sharply after 2020 and overtook that of the United States from 2022 onward, making it the fastest‐growing contributor in the most recent period. Citation impact showed a different ordering: China accumulated the most citations (9940), followed by the United States (6443), Australia (4414), South Korea (3625), and the United Kingdom (2747) (Figure 2C). Corresponding‐author country analysis (Figure 2D) indicated that China and the United States led in both single‐country and multiple‐country publications; overall, 17.0% of publications involved international collaboration (Figure S2).

FIGURE 2Geographical distribution and collaboration. (A) Country scientific production map; (B) annual production of leading countries over time; (C) top 10 most cited countries; (D) corresponding‐author countries by single‐country (SCP) and multiple‐country (MCP) publications.

**Figure:**
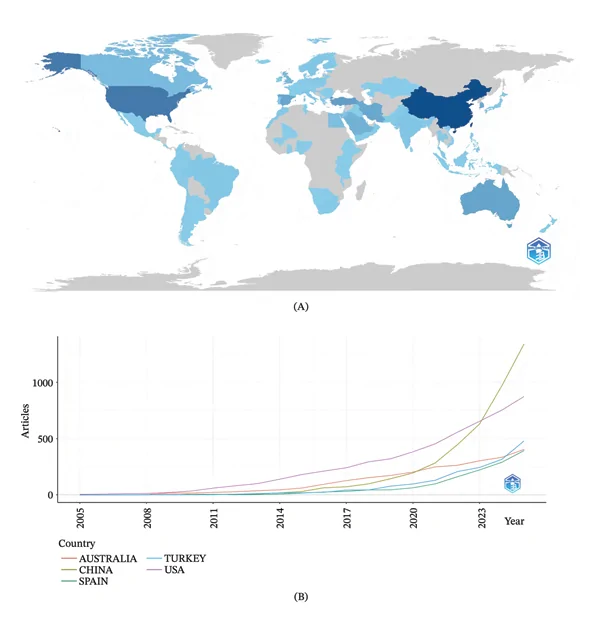

**Figure:**
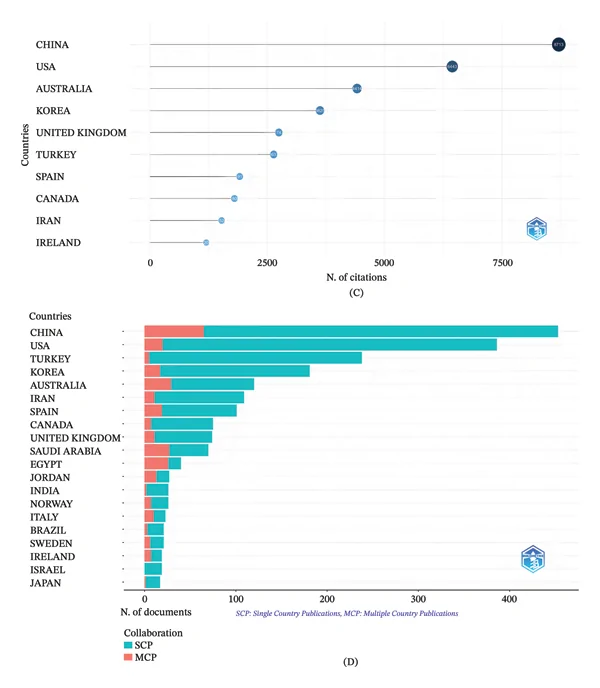

### 3.3. Journal Distribution and Citation Impact

The 2321 publications were distributed across 377 journals. Nurse Education Today was by far the most productive outlet (350 publications; 15.1% of the dataset), followed by BMC Nursing (162), Nurse Education in Practice (158), Journal of Nursing Education (87), and Journal of Professional Nursing (68) (Figure 3A). Bradford’s law identified a small core zone of journals accounting for a disproportionate share of the literature, led by Nurse Education Today (Figure 3B). Journal impact within the collection was highest for Nurse Education Today (h‐index 62), followed by Nurse Education in Practice (*h* = 28), Journal of Advanced Nursing (*h* = 25), BMC Nursing (*h* = 24), and Journal of Nursing Education (*h* = 24) (Figure 3C). Cumulative output dynamics (Figure 3D) showed sustained growth of Nurse Education Today throughout the period and a marked recent acceleration of BMC Nursing. Co‐citation analysis of cited journals identified Nurse Education Today (7050 citations), Nurse Education in Practice (2433), and Journal of Advanced Nursing (2123) as the most influential sources in the field’s reference base (in Table S9). The 10 most cited publications are listed with their construct anchors in Table S10.

FIGURE 3Journal distribution and impact. (A) Top 10 most productive sources; (B) core sources according to Bradford’s law; (C) top 10 sources by collection‐based h‐index; (D) cumulative source production dynamics.

**Figure:**
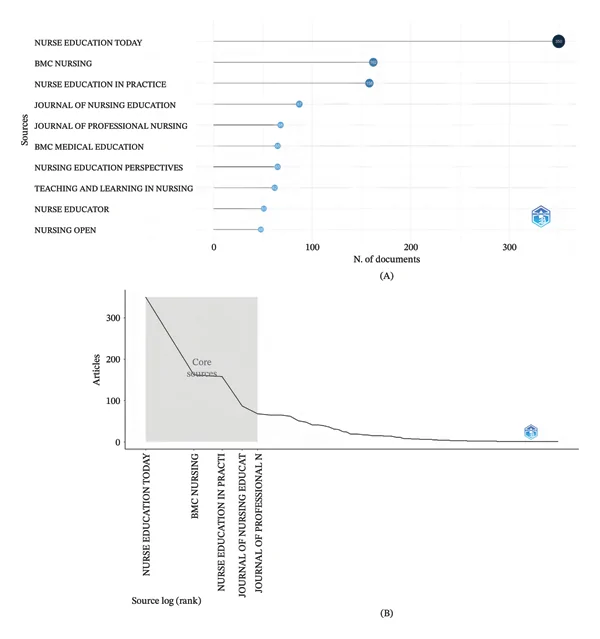

**Figure:**
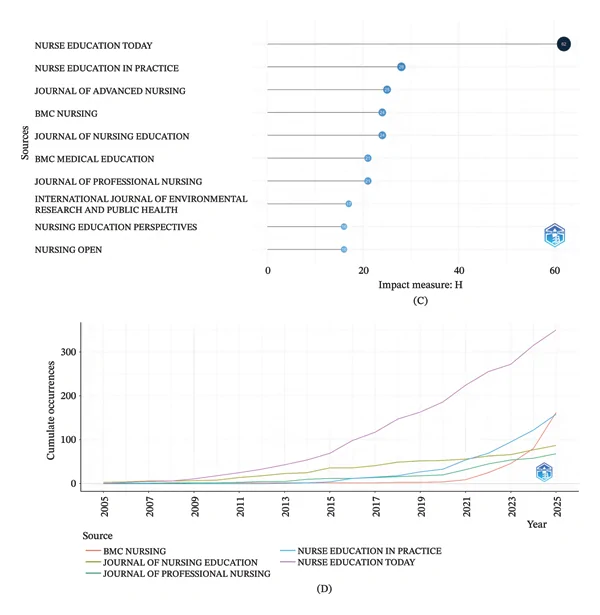

### 3.4. Institutional and Author Contributions

At the document level (each institution counted once per publication), the Egyptian Knowledge Bank—the national consortium platform through which Egyptian institutions publish—was the most frequent institutional affiliation (61 publications), followed by The Ohio State University (35), Alexandria University (29), the University of Texas System (27), and the University of Ha’il (26) (Figure 4A).

FIGURE 4Institutional and author contributions. (A) Top 10 most productive institutional affiliations (document‐level counts, WoS organizations‐enhanced names); (B) top 10 most productive authors after deterministic name disambiguation; (C) top 10 authors by collection‐based h‐index; (D) author collaboration network (41 nodes, 45 edges, 12 collaborative components; node size ∝ number of articles; edge width ∝ number of co‐authored articles; color = connected component). Isolated authors not shown.

**Figure:**
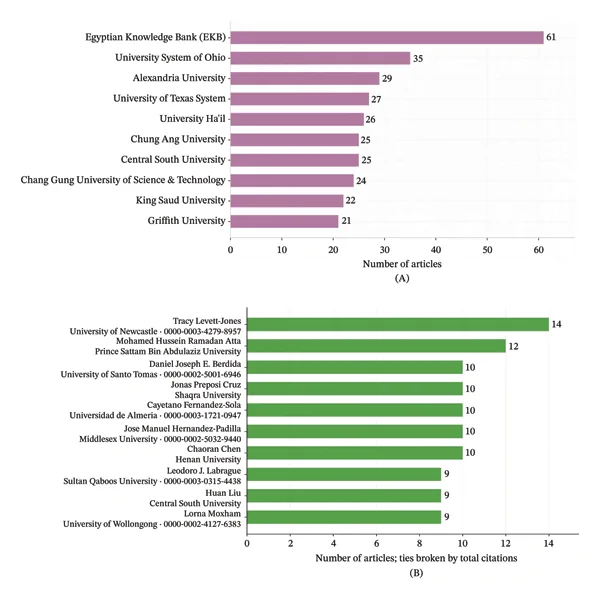

**Figure:**
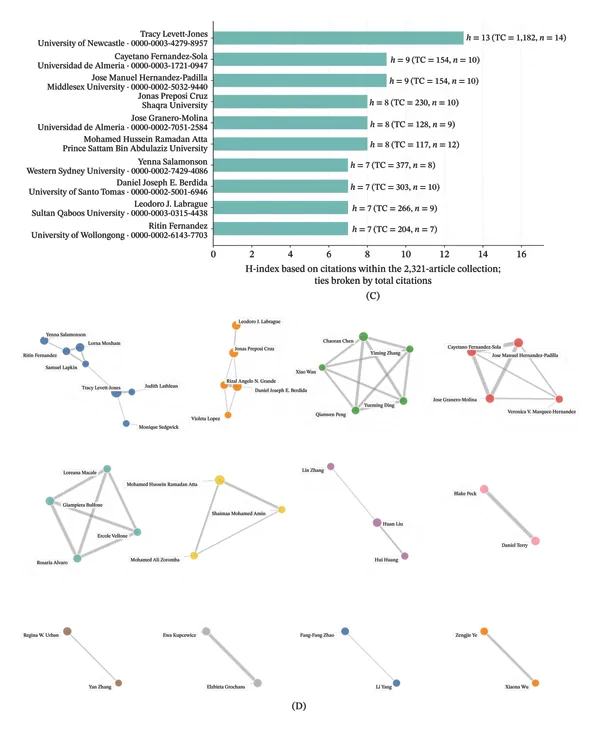

After deterministic author‐name disambiguation, 7728 unique authors contributed to the dataset (mean 4.23 co‐authors per document; 181 single‐authored publications). Tracy Levett‐Jones was the most productive author (14 publications), followed by Mohamed Hussein Ramadan Atta (12) and Daniel Joseph E. Berdida, Jonas Preposi Cruz, Cayetano Fernandez‐Sola, Jose Manuel Hernandez‐Padilla, and Chaoran Chen (10 each) (Figure 4B). Collection‐based h‐indices were highest for Levett‐Jones (*h* = 13), Fernandez‐Sola and Hernandez‐Padilla (*h* = 9 each), and Cruz, Granero‐Molina, and Atta (*h* = 8 each) (Figure 4C, Table S8). The author collaboration network (Figure 4D) comprised 41 nodes and 45 edges organized into 12 distinct collaborative components, indicating a field structured around relatively separate research groups rather than a single interconnected core.

### 3.5. Keyword Co‐Occurrence, Trend Topics, and Thematic Evolution

The most frequent keywords were nursing students (1100), self‐efficacy (380), nursing education (375), stress (190), resilience (186), simulation (162), emotional intelligence (160), professional identity (131), and COVID‐19 (130). Co‐occurrence clustering of the keyword network (50 nodes, 353 edges) identified eight thematic communities (Figure S3): (i) simulation‐based clinical education (simulation, self‐efficacy, critical thinking, communication, and clinical competence); (ii) resilience, stress, and coping (resilience, burnout, coping, psychological capital, self‐care); (iii) emotional intelligence and empathy; (iv) COVID‐19 and professional identity (COVID‐19, professional identity, psychological resilience, and nursing interns); (v) anxiety, depression, and mindfulness; (vi) clinical learning environment (clinical placements, belonging, empowerment, and satisfaction); (vii) learning approaches and academic performance (flipped classroom, gamification, online learning, motivation, and student engagement); and (viii) well‐being and health promotion (well‐being, life satisfaction, self‐esteem, and social support). Notably, artificial intelligence appeared in only 13 documents, and climate‐related content was not discernible as a cluster in the refined dataset.

Trend‐topic analysis (Figure 5) highlighted the recent rise of artificial intelligence, virtual reality, and empathy, whereas the recent acceleration of professional identity, online learning, and COVID‐19‐related themes was corroborated by the thematic evolution analysis (Figure S4). Thematic evolution across the three prespecified periods showed expansion from three communities in 2005–2013 (simulation/self‐efficacy teaching; stress and mental health; and clinical placements and belonging) to four in 2014–2020 (with differentiation of an emotional‐intelligence/mindfulness community and emergence of a professional‐identity/retention community) and five in 2021–2025 (the teaching community absorbing online learning, student engagement, and virtual reality; resilience/well‐being separating from stress/mental health; and a distinct COVID‐19/professional‐identity community) (Figure S5). The sensitivity analysis under alternative, data‐driven breakpoints (2012/2017) reproduced the same core domains and emergence order (see Methods), supporting the robustness of this narrative.

**FIGURE 5:**
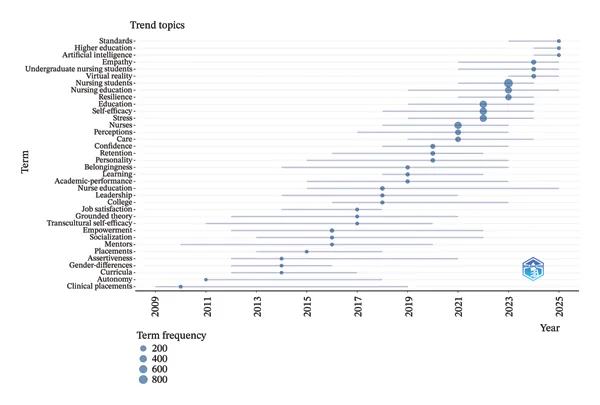
Trend topics in POP‐related nursing education research.

## 4. Discussion

This bibliometric analysis and narrative synthesis mapped global research on POP in nursing education from 2005 to 2025. Publication output grew rapidly (compound annual growth rate 27.2%), with marked acceleration after 2020. The United States and China were the two largest contributors, followed by Turkey, South Korea, and Australia, while Nurse Education Today, BMC Nursing, and Nurse Education in Practice formed the core journal zone. Keyword and thematic analyses showed a field anchored in stress, resilience, self‐efficacy, and simulation‐based education, with professional identity, online learning, and COVID‐19‐related themes emerging most strongly in recent years.

The POP framework is increasingly being applied or evaluated in nursing‐education research. Because POP connects individual psychological resources, relational learning environments, and organizational conditions, it may provide a useful integrative lens for this literature: rather than focusing only on distress, burnout, or educational deficits, POP‐oriented studies examine how nursing education systems can cultivate strengths, engagement, belonging, adaptability, and sustainable professional development. Whether this lens translates into improved educational outcomes remains an empirical question. Consistent with the synthesis framework described in the Methods, the Discussion below is organized around the themes retained by that synthesis: for each theme, we first summarize the bibliometric evidence, then examine the underlying eligible publications, and finally state the interpretation together with its evidential limits.

### 4.1. Growth of POP‐Related Research in Nursing Education

The increase in annual publication output indicates that POP‐related topics have moved from peripheral interest to a visible area of nursing education research [24, 25]. Growth after 2014 may reflect broader international attention to student well‐being, resilience, psychological capital, supportive learning climates, and strengths‐based education. The sharper increase after 2020 is likely associated with the combined effects of the COVID‐19 pandemic, disruptions in clinical placement, accelerated digital transformation, and heightened awareness of mental‐health needs among students and educators [26–28].

However, publication growth should not be interpreted as direct evidence that educational practice has improved. Bibliometric growth reflects scholarly attention, but it does not necessarily show that POP principles have been systematically implemented in curricula, faculty development, clinical supervision, or institutional policy. Future research should therefore examine how POP‐based strategies can be embedded into nursing education systems and evaluated using rigorous longitudinal, intervention, and mixed‐methods designs.

### 4.2. Geographical Distribution and Collaboration

The leading positions of China and the United States, together with substantial contributions from Turkey, South Korea, and Australia, suggest that POP‐related nursing education research is concentrated in countries with large nursing‐education systems and mature research infrastructures [29]. Two features deserve note. First, although the United States led in publication volume, China accumulated the highest total citations, consistent with its rapid post‐2020 expansion into highly visible outlets. Second, institutional productivity was headed by the Egyptian Knowledge Bank—the national consortium through which Egyptian universities publish—followed by The Ohio State University and Alexandria University, indicating growing participation from Middle Eastern and North African nursing‐education systems alongside the historically strong anglophone contributors.

At the same time, the uneven global distribution of publications highlights a limitation in the current knowledge base. Nursing education systems vary widely across cultural, economic, and healthcare contexts. Limited representation from Africa, South America, and some Asian regions may reduce the generalizability of existing evidence. Cross‐cultural and multicountry research is needed to understand how POP constructs such as resilience, psychological safety, empowerment, and engagement are shaped by educational systems, clinical placement models, faculty workload, and sociocultural expectations [16].

### 4.3. Established Themes: Stress, Resilience, Psychological Capital, and Well‐Being

Three established themes were retained at the robustness‐assessment stage of the synthesis because each rests on recurrent keyword communities and a substantial body of eligible publications; they are examined in turn below. A major established theme concerns stress, mental health, burnout, and coping among nursing students and educators. Nursing students experience academic workload, clinical uncertainty, interpersonal challenges, fear of making mistakes, and emotional exposure to patient suffering [30, 31]. Nurse educators and clinical instructors may experience role overload, emotional labor, and pressure to support students while meeting institutional expectations [32, 33]. These challenges are frequently cited in this literature as motivations for applying POP constructs in nursing education.

Resilience has emerged as one of the most visible constructs in this field. From a POP perspective, resilience is not merely an individual trait but a dynamic capacity shaped by learning environments, mentoring relationships, social support, and organizational culture. Resilience‐oriented education may help students and educators adapt to adversity, but resilience should not be used to shift responsibility from institutions to individuals [34, 35]. A stronger POP approach combines individual resilience‐building with organizational strategies, including psychological safety, manageable workload, supportive supervision, and inclusive educational leadership.

Psychological capital is another central construct. It includes hope, efficacy, resilience, and optimism and has been associated with motivation, engagement, adaptation, and performance in organizational and educational settings [36]. In nursing education, psychological capital may support students’ persistence, clinical confidence, professional identity formation, and readiness for transition to practice [37–39]. For educators, psychological capital may be related to teaching creativity, work engagement, and burnout prevention [40, 41]. The increasing appearance of psychological capital in recent years suggests that this construct may serve as a theoretical bridge between positive psychology, organizational behavior, and nursing education.

### 4.4. Professional Identity, Engagement, and Inclusive Educational Culture

Professional identity appeared as an important emerging theme. In nursing education, professional identity is shaped not only by curriculum content but also by role modeling, clinical placement experiences, peer relationships, faculty support, and perceptions of the nursing profession [42, 43]. POP contributes to this area by emphasizing meaning, strengths, belonging, and positive role development. Educational environments that foster psychological safety, recognition, and reflective learning may support students in developing a stable and positive professional identity [44, 45].

Work engagement and learning engagement are also important POP‐related outcomes [46]. Engagement reflects vigor, dedication, and absorption and is closely linked to motivation and persistence. In nursing education, engagement can be strengthened through active learning, authentic simulation, supportive feedback, autonomy‐supportive teaching, and meaningful clinical experiences [47, 48]. Faculty engagement is equally important because educators’ enthusiasm, creativity, and psychological well‐being influence the quality of teaching and mentoring [49]. Inclusive educational culture represents a broader theme that integrates psychological safety, equity, belonging, respectful communication, and supportive leadership. Nursing education increasingly involves diverse student populations, interprofessional learning environments, and complex clinical partnerships. POP provides a framework for examining how inclusive climates can reduce marginalization, increase participation, and promote well‐being. This is particularly relevant for students who face academic, cultural, financial, or psychological vulnerabilities.

### 4.5. Emerging Themes: Online Learning, Simulation Technologies, and Early Digital Signals

The emerging themes below derive from the trend‐topic and thematic‐evolution analyses and were confirmed against the eligible publication base during the exploration of relationships; bibliometric signals without such publication support, such as the former climate‐change conjecture, were excluded at the robustness‐assessment stage. Trend‐topic and thematic‐evolution analyses showed that online learning and technology‐supported pedagogies accelerated markedly after 2020, when the COVID‐19 pandemic forced rapid adoption of online learning, virtual simulation, and technology‐supported clinical education [50]. Virtual reality appeared in 46 documents, typically as immersive, psychologically safer environments in which students practice clinical decision‐making, communication, and technical skills without risk to patients [51]. From a POP perspective, such environments may enhance self‐efficacy, confidence, engagement, and adaptive learning when designed with appropriate feedback, scaffolding, and emotional support [50, 51]. Artificial intelligence remained peripheral in the refined dataset (13 documents) and should be interpreted as an early‐stage signal rather than an established theme. AI‐supported tools may assist nursing education through adaptive learning platforms, simulation analytics, automated feedback, and prediction of learning difficulties, but they also raise concerns about academic integrity, bias, data privacy, overreliance, and the erosion of interpersonal learning. POP can contribute to this discussion by emphasizing technology designs that support autonomy, competence, psychological safety, engagement, and ethical educational climates rather than simply increasing efficiency [52, 53].

Future research should evaluate how digital technologies influence POP‐related outcomes such as student confidence, engagement, psychological safety, faculty workload, and educational well‐being, moving beyond technology acceptance to examine whether digital tools create meaningful, inclusive, and emotionally supportive learning environments.

### 4.6. A Multilevel POP Framework for Nursing Education

The framework proposed in this subsection represents the theory‐development element of the synthesis: its levels were derived by mapping the confirmed themes onto positive‐psychology and organizational‐behavior theory rather than stipulated a priori. Based on the bibliometric and narrative findings, POP in nursing education can be understood through a multilevel framework. At the individual level, psychological capital, resilience, emotional intelligence, self‐efficacy, optimism, and well‐being support students’ and educators’ adaptive functioning. At the interpersonal level, mentoring, peer support, role modeling, respectful communication, and psychological safety shape learning relationships. At the educational level, active learning, simulation, reflective practice, feedback, and curriculum design influence engagement and competence development. At the organizational level, supportive leadership, inclusive culture, workload management, faculty development, and institutional policy determine whether positive psychological resources can be sustained. At the societal level, digital transformation, workforce shortages, and global health crises create new demands for nursing education.

This framework suggests that POP extends beyond individual positivity or resilience training. Instead, POP is portrayed in the synthesized literature as a multilevel approach linking psychological resources with organizational responsibility. Nursing schools and clinical education partners are positioned to create conditions that enable students and educators to flourish while maintaining high educational and professional standards.

### 4.7. Implications for Nursing Education, Research, and Policy

The findings have several implications for nursing education. Consistent with the descriptive nature of bibliometric evidence, these implications are offered as directions for consideration rather than as evidence that specific practices are effective. First, nursing curricula should include structured opportunities to develop psychological capital, resilience, reflective capacity, professional identity, and adaptive coping. These topics should be integrated into existing clinical, communication, leadership, and professional development courses rather than treated as optional wellness content. Second, faculty development programs should address educator well‐being, work engagement, positive leadership, psychological safety, and inclusive teaching practices. Supporting educators is essential because their well‐being and engagement influence the quality of student learning. Third, clinical education environments should be evaluated not only for technical learning opportunities but also for psychological safety, supportive supervision, respect, and belonging. Fourth, educational technology should be assessed in relation to learning quality and psychological outcomes, including confidence, engagement, ethical awareness, and emotional support. Finally, policy makers and institutional leaders should recognize that sustainable nursing education requires organizational conditions that protect well‐being and promote flourishing at both student and faculty levels.

### 4.8. Strengths and Limitations

This study has several strengths. Methodologically, the search strategy was validated against a frozen seed set (verified recall 96%) and selected through a prespecified precision duel between candidate tiers; screening was conducted double‐blind under a piloted codebook with full adjudication of disagreements, and an independent audit of excluded records found a false‐exclusion rate of 1.5%. Author‐level analyses used deterministic name disambiguation with a bounded residual collision risk (≤ 0.27%). Substantively, the study integrates publication trends, country and institutional contributions, journal analysis, author networks, keyword co‐occurrence, thematic evolution, and narrative synthesis over 2 decades, and its thematic narrative was robust to alternative period segmentations.

Several limitations should be acknowledged. First, the analysis was based only on WoSCC records searched on 6 August 2026; publications indexed exclusively in Scopus, PubMed, CINAHL, ERIC, or regional databases, and publications appearing after the search date, were not covered. Second, the English‐language restriction may have excluded relevant studies in other languages. Third, bibliometric results are influenced by database coverage, indexing practices, citation accumulation, and keyword variability; although author names were deterministically disambiguated, a residual collision risk of up to 0.27% cannot be excluded. Fourth, citation counts favor older publications and do not directly reflect methodological quality or practical significance. Fifth, the three‐period thematic segmentation was prespecified for descriptive purposes and was not subjected to formal change‐point inference; a sensitivity analysis under data‐driven breakpoints nonetheless reproduced the same thematic narrative. Finally, the narrative synthesis was interpretive and did not assess intervention effectiveness or risk of bias at the individual‐study level.

### 4.9. Future Directions

Future research should advance in several directions. First, theory‐driven intervention studies are needed to test whether POP‐based educational strategies improve resilience, psychological capital, engagement, professional identity, and well‐being among nursing students and educators. Second, longitudinal designs should examine how POP‐related resources develop across nursing programs and during the transition to professional practice. Third, cross‐cultural research should explore how POP constructs operate in diverse educational systems and clinical learning environments. Fourth, implementation studies should examine how organizational policies, faculty development, mentoring, and workload management can sustain positive educational climates. Fifth, as online learning, simulation technologies, and artificial intelligence diffuse into nursing education, research should examine not only technical effectiveness but also psychological safety, equity, engagement, and ethical implications.

## 5. Conclusion

This bibliometric analysis and narrative synthesis shows that research on POP in nursing education expanded rapidly from 2005 to 2025, reaching 2321 publications with a compound annual growth rate of 27.2%. The field has evolved from early concerns about educational methods, stress, and workplace challenges toward a broader agenda that foregrounds resilience, psychological capital, self‐efficacy, professional identity, engagement, and supportive learning climates, with online learning and simulation technologies accelerating after 2020. The United States and China were the largest contributors, and Nurse Education Today, BMC Nursing, and Nurse Education in Practice were the major outlets. The synthesized literature presents POP as a multilevel framework connecting individual psychological resources with relational, educational, and organizational conditions in nursing education. Future research should test this framework through rigorous intervention, longitudinal, and implementation studies to establish whether the POP applications described in this literature lead to measurable improvements in educational quality and the well‐being of nursing students and educators.

## Author Contributions

Haozhang Huang and Xuting Chen conceived and designed the study. Ming Chen performed the bibliometric analysis and contributed to data interpretation. Shuibing Cai and Haozhang Huang independently and in double control conducted a secondary screening based on the inclusion criteria. Wanqi Ou, Ming Chen, Shuibing Cai, and Qiaoyan Guan contributed to data curation and manuscript drafting. Haiyi Liang, Ying Cao, and Yueya Wang contributed to literature interpretation and manuscript revision. Haozhang Huang and Xuting Chen supervised the study and critically revised the manuscript.

## Funding

This study was supported by the Traditional Chinese Medicine Bureau of Guangdong Province (20262003).

## Disclosure

All authors read and approved the final manuscript.

## Conflicts of Interest

The authors declare no conflicts of interest.

## Supporting Information

Additional supporting information can be found online in the Supporting Information section.

## Supporting information


**Supporting Information** Figure S1. PRISMA 2020 flow diagram of record identification, screening, and inclusion. Figure S2. Country collaboration network. Figure S3. Keyword co‐occurrence network (50 nodes, 353 edges; Louvain clustering). Figure S4. Thematic evolution of keyword communities across the three prespecified periods (2005–2013, 2014–2020, 2021–2025). Figure S5. Thematic map (motor, basic, niche, and emerging/declining themes). Table S1. Completed BIBLIO checklist for reporting bibliometric reviews of the biomedical literature, with the manuscript location of each item. Table S2. Three‐tier search strategies for POP‐related nursing‐education research in the Web of Science Core Collection. Table S3. Seed‐set composition and recall verification. Table S4. Formal recall against WoS exports (DOI/title dual matching, normalized title similarity ≥ 0.90). Table S5. Precision‐duel results. Table S6. Eligibility codes. Table S7. Batch‐level double‐blind screening statistics. Table S8. Top 10 most productive authors after disambiguation. Table S9. Top 10 cited sources in the reference base. Table S10. The top 10 cited publications about POP in nurse education.

## Data Availability

The data analyzed in this study were retrieved from the Web of Science Core Collection. The data that support the findings of this study also are available from the corresponding authors upon reasonable request.

## References

[1] Küçük Yüceyurt N. and Kaya H. , Curriculum/Program Development in Nursing Education: A Systematic Review, BMC Nursing. (2026) 25, no. 1, 10.1186/s12912-026-04342-3. PMC12973920 41652574

[2] Mudzi P. Y. , Mabizela S. , and Bruce J. , Nurse Leader Perspectives of Organisational Capacity for Higher Education: Implications for Nursing Education Policy, BMC Nursing. (2025) 24, no. 1, 10.1186/s12912-025-03312-5. PMC12135258 40468314

[3] Arieli D. , Emotional Work and Diversity in Clinical Placements of Nursing Students, Journal of Nursing Scholarship. (2013) 45, no. 2, 192–201, 10.1111/jnu.12020. 23462103

[4] Pearson M. C. and Shumway J. , Exploring the Impact on Faculty of the American Association of Colleges of Nursing’s the Essentials: Core Competencies for Professional Nursing Education, Journal of Professional Nursing. (2025) 57, 139–147, 10.1016/j.profnurs.2025.02.003. 40074377

[5] Livesay K. , Walter R. , Petersen S. , Abdolkhani R. , Zhao L. , and Butler-Henderson K. , Challenges and Needs in Digital Health Practice and Nursing Education Curricula: Gap Analysis Study, JMIR Medical Education. (2024) 10, 10.2196/54105. PMC11414417 39269365

[6] Peng X. , Yang L. , Yang S. , Wang X. , and Song W. , Effects of Clinical Learning Environment on the Professional Identity of Chinese Nursing Students: Multiple Mediating Effects of Learning Engagement and Stress-Related Growth, BMC Nursing. (2025) 24, no. 1, 10.1186/s12912-025-03641-5. PMC12607047 41219932

[7] Seligman M. E. , Steen T. A. , Park N. , and Peterson C. , Positive Psychology Progress: Empirical Validation of Interventions, American Psychologist. (2005) 60, no. 5, 410–421, 10.1037/0003-066x.60.5.410. 16045394

[8] Seligman M. E. P. , Positive Psychology: A Personal History, Annual Review of Clinical Psychology. (2019) 15, 1–23, 10.1146/annurev-clinpsy-050718-095653. 30525996

[9] Martín-Del-Río B. , Neipp M. C. , García-Selva A. , and Solanes-Puchol A. , Positive Organizational Psychology: A Bibliometric Review and Science Mapping Analysis, International Journal of Environmental Research and Public Health. (2021) 18, no. 10, 10.3390/ijerph18105222. PMC8157200 34068995

[10] Zhang J. , Rehman S. , Addas A. , and Ahmad J. , Influence of Work-Life Balance on Mental Health Among Nurses: The Mediating Role of Psychological Capital and Job Satisfaction, Psychology Research and Behavior Management. (2024) 17, 4249–4262, 10.2147/prbm.s497305. PMC11646404 39679316

[11] Ma H. , Zhu X. , Huang J. , Zhang S. , Tan J. , and Luo Y. , Assessing the Effects of Organizational Support, Psychological Capital, Organizational Identification on Job Performance Among Nurses: A Structural Equation Modeling Approach, BMC Health Services Research. (2023) 23, no. 1, 10.1186/s12913-023-09705-z. PMC10375763 37501166

[12] Flinkman M. , Rudman A. , Pasanen M. , and Leino-Kilpi H. , Psychological Capital, Grit and Organizational Justice as Positive Strengths and Resources Among Registered Nurses: A Path Analysis, Nursing Open. (2023) 10, no. 8, 5314–5327, 10.1002/nop2.1769. PMC10333875 37128977

[13] Ruiz-Fernández M. D. , Alcaraz-Córdoba A. , López-Rodríguez M. M. , Fernández-Sola C. , Granero-Molina J. , and Hernández-Padilla J. M. , The Effect of Home Visit Simulation on Emotional Intelligence, Self-Efficacy, Empowerment, and Stress in Nursing Students. A Single Group Pre-Post Intervention Study, Nurse Education Today. (2022) 117, 10.1016/j.nedt.2022.105487. 35926341

[14] Neufeld A. , Orsini C. , and Imafuku R. , A Leadership Guide to Applying the Job Demands-Resources (JD-R) Theory for Faculty Engagement and Burnout Prevention in Health Professions Education: AMEE Guide No. 194, Medical Teacher. (2026) 1–12, 10.1080/0142159x.2026.2649948. 41984423

[15] Boamah S. A. , Alumona C. J. , Onyeso O. K. , and Awosoga O. A. , Institutional and Personal Determinants of Nursing Educators’ Job Satisfaction and Turnover Intention: A Cross-Sectional Study, BMC Nursing. (2024) 23, no. 1, 10.1186/s12912-024-02245-9. PMC11346278 39183300

[16] van Rensburg G. H. , Matahela V. E. , and Horton-Deutsch S. , Providing Healthy Work Environments Through Facilitation of Nurse Educators’ Self-Leadership in Academic Settings: A Qualitative Explorative Study, SAGE Open Nursing. (2025) 11, 10.1177/23779608251366955. PMC12332250 40786832

[17] Page M. J. , McKenzie J. E. , Bossuyt P. M. et al., The PRISMA 2020 Statement: An Updated Guideline for Reporting Systematic Reviews, BMJ. (2021) 372. 10.1136/bmj.n71 PMC8005924 33782057

[18] Rethlefsen M. L. , Kirtley S. , Waffenschmidt S. et al., PRISMA-S: An Extension to the PRISMA Statement for Reporting Literature Searches in Systematic Reviews, Systematic Reviews. (2021) 10, no. 1. 10.1186/s13643-020-01542-z PMC7839230 33499930

[19] Montazeri A. , Mohammadi S. , P M. H. , Ghaemi M. , Riazi H. , and Sheikhi-Mobarakeh Z. , Preliminary Guideline for Reporting Bibliometric Reviews of the Biomedical Literature (BIBLIO): A Minimum Requirements, Systematic Reviews. (2023) 12, no. 1, 10.1186/s13643-023-02410-2. PMC10722750 38102710

[20] Pranckutė R. , Web of Science (WoS) and Scopus: The Titans of Bibliographic Information in Today’s Academic World, Publications. (2021) 9, no. 1, 10.3390/publications9010012.

[21] Aria M. and Cuccurullo C. , Bibliometrix: An R-Tool for Comprehensive Science Mapping Analysis, Journal of Informetrics. (2017) 11, no. 4, 959–975, 10.1016/j.joi.2017.08.007.

[22] Blondel V. D. , Guillaume J.-L. , Lambiotte R. , and Lefebvre E. , Fast Unfolding of Communities in Large Networks, Journal of Statistical Mechanics: Theory and Experiment. (2008) 2008, no. 10, 10.1088/1742-5468/2008/10/p10008.

[23] Popay J. , Roberts H. , Sowden A. J. et al., Guidance on the Conduct of Narrative Synthesis in Systematic Reviews, ESRC Methods Programme.

[24] Han J. W. , Kang K. I. , and Joung J. , Enhancing Happiness for Nursing Students Through Positive Psychology Activities: A Mixed Methods Study, International Journal of Environmental Research and Public Health. (2020) 17, no. 24, 10.3390/ijerph17249274. PMC7762976 33322448

[25] Li H. , Glecia A. , Opoku-Mensah F. , Labrecque M. E. , and Petrucka P. , Integrating Psychological Well-Being and Self-Care Behaviors into Undergraduate Nursing Education Curriculum: A Scoping Review, Nursing Education Perspectives. (2025) 46, no. 6, E42–e7, 10.1097/01.nep.0000000000001466. 41021743

[26] Carolan C. , Davies C. L. , Crookes P. , McGhee S. , and Roxburgh M. , COVID 19: Disruptive Impacts and Transformative Opportunities in Undergraduate Nurse Education, Nurse Education in Practice. (2020) 46, 10.1016/j.nepr.2020.102807. PMC7255089 32502757

[27] Head M. L. , Acosta S. , Bickford E. G. , and Leatherland M. A. , Impact of COVID-19 on Undergraduate Nursing Education: Student Perspectives, Academic Medicine. (2022) 97, no. 3s, S49–s54, 10.1097/acm.0000000000004530. PMC8855759 34789659

[28] O′Keefe R. and Auffermann K. , Exploring the Effect of COVID-19 on Graduate Nursing Education, Academic Medicine. (2022) 97, no. 3s, S61–s5, 10.1097/ACM.0000000000004537. PMC8855768 34817402

[29] Aiken L. H. , Sloane D. M. , Bruyneel L. et al., Nurse Staffing and Education and Hospital Mortality in Nine European Countries: A Retrospective Observational Study, Lancet. (2014) 383, no. 9931, 1824–1830, 10.1016/s0140-6736(13)62631-8. PMC4035380 24581683

[30] Christianson J. , Malin K. , Leiberg J. , Grabert L. , Moser S. , and Zemlak J. , Educating to Exhaustion: Intention to Leave Among US Full-Time Nursing Faculty, Nursing Education Perspectives. (2026) 47, no. 2, 86–95, 10.1097/01.nep.0000000000001492. PMC12911495 41701856

[31] Wong K. P. , Zhang B. , Xie Y. J. et al., Impacts of Job Demands on Turnover Intention Among Registered Nurses in Hong Kong Public Hospitals: Exploring the Mediating Role of Burnout and Moderating Effect of Pay Level Satisfaction, Journal of Nursing Management. (2024) 2024, no. 1, 10.1155/2024/3534750. PMC11918518 40224872

[32] Skaria R. , Whitehead D. , Leach L. , and Walshaw M. , Experiences of Overseas Nurse Educators Teaching in New Zealand, Nurse Education Today. (2019) 81, 7–12, 10.1016/j.nedt.2019.05.032. 31295662

[33] Cummins A. and Gilkison A. , Insights and Innovations in Midwifery Education, the Past, Present and Future, Women and Birth. (2023) 36, no. 2, 141–142, 10.1016/j.wombi.2022.12.009. 36599783

[34] Schwartz-Mette R. A. , Sapp M. , and Strout K. , Online and In-Person Mindfulness-Based Stress Reduction (MBSR) Improves Stress Resilience in Nursing Education, Worldviews on Evidence-Based Nursing. (2025) 22, no. 3, 10.1111/wvn.70031. PMC12066802 40350560

[35] Wei H. , Roberts P. , Strickler J. , and Corbett R. W. , Nurse Leaders’ Strategies to Foster Nurse Resilience, Journal of Nursing Management. (2019) 27, no. 4, 681–687, 10.1111/jonm.12736. 30449038

[36] Zhou J. , Yang Y. , Qiu X. et al., Serial Multiple Mediation of Organizational Commitment and Job Burnout in the Relationship Between Psychological Capital and Anxiety in Chinese Female Nurses: A Cross-Sectional Questionnaire Survey, International Journal of Nursing Studies. (2018) 83, 75–82, 10.1016/j.ijnurstu.2018.03.016. 29702357

[37] Zheng N. , Zhang T. , He X. F. , and Zhu X. Q. , Decent Work Perception and Its Relationship With Work-Related Flow and Psychological Capital Among Nurses: A Cross-Sectional Study, Journal of Advanced Nursing. (2026) 82, no. 4, 2947–2958, 10.1111/jan.17100. 40538106

[38] Zhou H. , Peng J. , Wang D. et al., Mediating Effect of Coping Styles on the Association Between Psychological Capital and Psychological Distress Among Chinese Nurses: A Cross-Sectional Study, Journal of Psychiatric and Mental Health Nursing. (2017) 24, no. 2-3, 114–122, 10.1111/jpm.12350. 28233374

[39] Hao J. , Wu D. , Liu L. , Li X. , and Wu H. , Association Between Work-Family Conflict and Depressive Symptoms Among Chinese Female Nurses: The Mediating and Moderating Role of Psychological Capital, International Journal of Environmental Research and Public Health. (2015) 12, no. 6, 6682–6699, 10.3390/ijerph120606682. PMC4483724 26075725

[40] Jung S. , Han K. , and Kim K. , Individual-Level Positive Psychological Capital that Enhances Managers’ Transformational Leadership Effectiveness on Nurse Career Success, International Nursing Review. (2024) 71, no. 4, 977–984, 10.1111/inr.12958. 38477826

[41] Jin M. , Zhang Y. , Wang F. et al., A Cross Sectional Study of the Impact of Psychological Capital on Organisational Citizenship Behaviour Among Nurses: Mediating Effect of Work Engagement, Journal of Nursing Management. (2022) 30, no. 5, 1263–1272, 10.1111/jonm.13609. 35338531

[42] Guo Y. J. , Yang L. , Ji H. X. , and Zhao Q. , Caring Characters and Professional Identity Among Graduate Nursing Students in China-A Cross Sectional Study, Nurse Education Today. (2018) 65, 150–155, 10.1016/j.nedt.2018.02.039. 29579567

[43] Yang L. , Li S. N. , Wang Q. , Pan S. , and Zhao W. , Unveiling the Relationships of Professional Identity, Values and Communication Competence in Nursing Students’ Clinical Practice Behaviours: A Cross-Sectional Study and Mediation Model Analysis, Nursing Open. (2025) 12, no. 7, 10.1002/nop2.70270. PMC12238724 40629945

[44] Amin S. M. , El-Ashry A. M. , Karim N. et al., Shaping Competence Through Identity: The Mediating Role of Professional Identity in the Relationship Between Clinical Learning Environment and Nursing Competence Among Nursing Students, Nurse Education Today. (2026) 157, 10.1016/j.nedt.2025.106905. 41237604

[45] Iacobucci T. A. , Daly B. J. , Lindell D. , and Griffin M. Q. , Professional Values, self-esteem, and Ethical Confidence of Baccalaureate Nursing Students, Nursing Ethics. (2013) 20, no. 4, 479–490, 10.1177/0969733012458608. 23166146

[46] McCaughey D. , McGhan G. , Walsh E. M. , Rathert C. , and Belue R. , The Relationship of Positive Work Environments and Workplace Injury: Evidence From the National Nursing Assistant Survey, Health Care Management Review. (2014) 39, no. 1, 75–88, 10.1097/hmr.0b013e3182860919. 23416788

[47] Turjuman F. and Alilyyani B. , Emotional Intelligence Among Nurses and Its Relationship With Their Performance and Work Engagement: A Cross-Sectional Study, Journal of Nursing Management. (2023) 2023, 10.1155/2023/5543299. PMC11919067 40225659

[48] Gao X. , Zhou Y. , Xu X. , Yuan R. , Zheng Y. , and Yun R. , Relationships Between Organizational Support, Emotional Intelligence and Work Engagement Among Chinese Nurses: A Correlation Study, Nursing Open. (2024) 11, no. 10, 10.1002/nop2.70034. PMC11474886 39404068

[49] Cao T. , Huang X. , Wang L. et al., Effects of Organisational Justice, Work Engagement and Nurses’ Perception of Care Quality on Turnover Intention Among Newly Licensed Registered Nurses: A Structural Equation Modelling Approach, Journal of Clinical Nursing. (2020) 29, no. 13-14, 2626–2637, 10.1111/jocn.15285. 32279372

[50] Huai P. , Li Y. , Wang X. , Zhang L. , Liu N. , and Yang H. , The Effectiveness of Virtual Reality Technology in Student Nurse Education: A Systematic Review and Meta-Analysis, Nurse Education Today. (2024) 138, 10.1016/j.nedt.2024.106189. 38603830

[51] Ota Y. , Aikawa G. , Nishimura A. , Kawashima T. , Imanaka R. , and Sakuramoto H. , Effects of Educational Methods Using Extended Reality on Pre-Registration Nursing Students’ Knowledge, Skill, Confidence, and Satisfaction: A Systematic Review and Meta-Analysis, Nurse Education Today. (2024) 141, 10.1016/j.nedt.2024.106313. 39042984

[52] El-Sayed B. K. M. , El-Sayed A. A. I. , Alsenany S. A. , and Asal M. G. R. , The Role of Artificial Intelligence Literacy and Innovation Mindset in Shaping Nursing Students’ Career and Talent Self-Efficacy, Nurse Education in Practice. (2025) 82, 10.1016/j.nepr.2024.104208. 39637623

[53] Gürdap Z. and Öner U. , Nurses’ Attitudes Toward Artificial Intelligence Applications and Their Clinical Decision-Making Competence: A Cross-Sectional Study, Nurse Education Today. (2026) 160, 10.1016/j.nedt.2026.107014. 41619448

