# Supplementary material for: Positive Organizational Psychology in Nursing Education: A Bibliometric Analysis and Narrative Review (2005–2025)
Source: J Nurs Manag. 2026 Oct 17;2026:9914092. doi: 10.1155/jonm/9914092 (PMC13675943; doi:10.1155/jonm/9914092)
Supplement: Supplementary file 1 [file JONM-2026-9914092-s001.docx]

# 1. Record identification, screening, and inclusion (PRISMA 2020)

The search of the Web of Science Core Collection (executed on 6 August 2026; publication window 1 January 2005 to 31 December 2025; editions: SCI-EXPANDED, SSCI, A&HCI, ESCI) returned 13,658 records. After duplicate removal (n=13) and pre-filtering (n=1,284: 311 non-English; 372 non-research document types; 366 conference proceedings or book chapters; 15 retracted; 220 early-access records formally published in 2026 and therefore outside the window), 12,361 records entered independent double-blind title/abstract screening; 10,029 were excluded, leaving 2,332 records at the eligibility stage. Of these, 16 records of uncertain eligibility were assessed at full text; 11 were excluded (nine for insufficient POP-construct evidence, two for population mismatch), including four records re-assessed at full-text level by the principal investigator during revision. The final dataset comprised 2,321 publications.

Figure S1. PRISMA 2020 flow diagram of record identification, screening, and inclusion. (Abbreviations: PY: publication years)

# 2. BIBLIO checklist

**Table S1. Completed BIBLIO checklist for reporting bibliometric reviews of the biomedical literature, with the location of each item in this manuscript.**

| **Section/topic** | **Item no** | **Checklist item** | **Reported in** |
| --- | --- | --- | --- |
| Title | 1 | Identification: identify the report as a bibliometric review/analysis in the title. | Title (page 1): "a bibliometric analysis combined with a narrative review" |
| Title | 2 | Issues/topics: state the key issues or topics covered and the time period of the literature. | Title and Abstract (POP in nursing education, 2005–2025) |
| Abstract | 3 | Structured summary: provide a structured summary including background, methods, results, and conclusions. | Abstract (page 2) |
| Introduction | 4 | Justification/rationale: summarize the existing knowledge and relevant background information that justifies the review. | Introduction, opening paragraphs |
| Introduction | 5 | Objectives: state the objectives or questions the review addresses. | Introduction, final paragraph |
| Methods | 6 | Search engines and data sources: name the databases/search engines used and the time span searched. | Methods – Data source (Web of Science Core Collection; search executed 6 August 2026) |
| Methods | 7 | Search strategy: present the full search strategy, including keywords, date of search, language, and document type. | Methods – Search strategy: a validated three-tier design; Table S2 |
| Methods | 8 | Time period: state the period covered by the review and justify the choice. | Methods – Data source and Eligibility criteria (2005–2025) |
| Methods | 9 | Eligibility criteria: specify the inclusion and exclusion criteria. | Methods – Eligibility criteria, screening, and selection audit; Table S6 |
| Methods | 10 | Data refinement: describe the selection procedure, including deduplication and screening steps. | Methods – Eligibility criteria, screening, and selection audit; Figure S1 (PRISMA flow diagram) |
| Methods | 11 | Quality assessment (optional): describe any procedure for assessing the quality of included publications. | Not applicable (optional item); rigour was addressed through double-blind screening, an exclusion audit, and the robustness assessment (Methods – Narrative synthesis) |
| Methods | 12 | Data synthesis: describe the methods used to summarize, handle, and synthesize the data, including any flow diagram. | Methods – Bibliometric analysis; Methods – Narrative synthesis; Figure S1 |
| Results | 13 | Descriptive findings/statistics: report the numbers of citations, publications, authors, journals, and institutions; include a flow diagram. | Results – General characteristics and publication growth; Figure S1 |
| Results | 14 | Schematic map and trend: present maps and trends produced with the analysis software. | Results – Keyword co-occurrence, trend topics, and thematic evolution; Figures 2–5; Figures S2–S5 |
| Results | 15 | Presentation approach: present the results in tables and figures organized by subtitle. | Results (subsections with Figures 1–5); Tables S8–S10 |
| Results | 16 | Synthesis of findings: identify gaps and, where possible, propose a model or hypothesis. | Results – Keyword co-occurrence, trend topics, and thematic evolution; Discussion – A multilevel POP framework for nursing education |
| Discussion | 17 | Summary of evidence: summarize the main findings of the review. | Discussion – Principal findings |
| Discussion | 18 | Interpretation: interpret the findings in the context of the existing evidence. | Discussion – Established themes; Emerging themes; A multilevel POP framework |
| Discussion | 19 | Strengths and limitations: discuss the strengths and limitations of the review. | Discussion – Strengths and limitations |
| Discussion | 20 | Conclusion(s): provide a general interpretation of the results and directions for future research. | Discussion – Future directions; Conclusion |

# 3. Three-tier search strategies

**Table S2. Three-tier search strategies for POP-related nursing-education research in the Web of Science Core Collection.**

| **Tier / facet** | **Search field** | **Query** | **Final combination / status** |
| --- | --- | --- | --- |
| Shared context facet #1 | TS (Topic) | TS=("nursing student*" OR "student nurs*" OR "nursing education" OR "nurse* education" OR "nurse educator*" OR "nursing educator*" OR "nursing facult*" OR "nursing school*" OR "school* of nursing" OR "college* of nursing" OR "undergraduate nurs*" OR "baccalaureate nurs*" OR "pre-registration nurs*" OR "preregistration nurs*" OR "pre-licensure nurs*" OR "prelicensure nurs*" OR "graduate nursing" OR "nursing program*" OR "nursing programme*" OR "nursing curricul*" OR "nursing train*" OR "nursing preceptor*" OR "nursing placement*") | Identical in all tiers; held constant so that recall/precision differences are attributable solely to the construct facet |
| Tier 1 (strict) — construct facet #2 | TI (Title) | TI=("positive psychology" OR "positive organizational" OR "psychological capital" OR psycap OR resilien* OR hardiness OR mindful* OR meditat* OR "well-being" OR wellbeing OR "well being" OR "self-compassion" OR "self compassion" OR "self-care" OR "self care" OR "professional identity" OR "emotional intelligence" OR "self-efficacy" OR "self efficacy" OR engag* OR motivat* OR grit OR flourish* OR thriv* OR empower* OR happiness OR happy OR "life satisfaction" OR optimis* OR hope* OR "strengths-based" OR "strength-based" OR "post-traumatic growth" OR "posttraumatic growth") | Final: #1 AND #2 Highest precision; not selected |
| Tier 2 (moderate) — construct facet #3 | TS (Topic) | TS=("positive psychology" OR "positive organizational" OR "psychological capital" OR psycap OR resilien* OR hardiness OR mindful* OR meditat* OR "well-being" OR wellbeing OR "well being" OR "self-compassion" OR "self compassion" OR "self-care" OR "self care" OR "professional identity" OR "emotional intelligence" OR "self-efficacy" OR "self efficacy" OR engag* OR motivat* OR grit OR flourish* OR thriv* OR empower* OR happiness OR happy OR "life satisfaction" OR optimis* OR hope* OR "strengths-based" OR "strength-based" OR "post-traumatic growth" OR "posttraumatic growth" OR "positive emotion*" OR "positive affect" OR "sense of coherence" OR belonging* OR "self-esteem" OR "self-determination" OR "intrinsic motivation" OR "personal growth" OR "meaning in life" OR gratitude OR "compassion satisfaction" OR "academic satisfaction") | Final: #1 AND #3 SELECTED |
| Tier 3 (broad) — construct facet #4 | TS (Topic) | TS=(<Tier 2 vocabulary> OR cope OR coping OR stress* OR burnout* OR gamif* OR "serious game*" OR "escape room" OR "academic success" OR retention OR persist* OR (positive NEAR/2 (psychol* OR emotion* OR affect OR attitude* OR learning OR experience*))) | Final: #1 AND #4 Recall ceiling; tested and rejected by the pre-specified decision rule |

Design principles. (i) the nursing-education context facet (#1) was held identical across tiers so that recall/precision differences are attributable solely to the POP construct facet; (ii) no NOT operators were used—exclusions were made at the screening stage and remained auditable; (iii) the construct vocabulary was derived empirically from the full texts of a frozen seed set of 50 verified eligible publications and the indexing terms of six peer-reviewed source reviews.

Note. High-noise deficit vocabulary (cope, coping, stress*, burnout*) was deliberately excluded from the selected Tier 2 and used only in the recall-ceiling tier, which was tested and rejected by the pre-specified decision rule.

# 4. Frozen seed set and recall verification

Seed-set construction: candidates were drawn from six peer-reviewed systematic/integrative/scoping reviews, technically filtered (journal articles, 2005–2025, English, WoSCC-indexed), independently re-screened in duplicate against the eligibility criteria, and stratified by construct branch and period; 50 seeds were selected from a qualified pool of 77 and frozen before any search testing. One seed not yet indexed by WoSCC was replaced per the pre-specified rule. All 50 seeds passed full-text eligibility re-verification.

**Table S3. seed-set composition and recall verification**

| **Item** | **Detail** |
| --- | --- |
| Construct branches | Engagement/motivation 15 items;  mindfulness 14 items;  resilience 13 items;  well-being/self-care 7 items;  professional identity 1 item |
| Period distribution | 2005–2014: 14 items;  2015–2019: 16 items;  2020–2025: 20 items |
| Source reviews | 6 peer-reviewed reviews (single source ≤30%) |
| WoSCC verification | 49/50 retrievable; 1 pre-specified replacement (journal not yet indexed) |
| Full-text re-verification | 50/50 confirmed eligible (bibliographic match + eligibility evidence) |

**Table S4. Formal recall against WoS exports (DOI/title dual matching, normalized title similarity ≥0.90)**

| **Tier** | **Field scope** | **Verified seed recall** | **Decision (threshold ≥90%)** |
| --- | --- | --- | --- |
| Tier 1 (strict) | TI | 31/50 = 62% | Rejected — structurally too strict: engagement/motivation studies name only the intervention format (games, VR, simulation) in their titles |
| Tier 2 (moderate) | TS | 48/50 = 96% | Qualified |
| Tier 3 (broad) | TS extended | 49/50 = 98% | Qualified |

# 5. Precision duel between qualifying tiers

A stratified random sample of 300 records per tier (100 per period 2005–2014/2015–2019/2020–2025) was double-coded independently by two screeners. After codebook calibration (version 3) and principal-investigator arbitration of 42 residual disagreements, agreement was 93.0% (558/600) and Cohen's κ was 0.811, exceeding the pre-specified 0.75 threshold. The pre-specified decision rule selected the qualifying tier with the highest precision.

**Table S5. precision-duel results**

| **Statistic** | **Tier 2 (moderate)** | **Tier 3 (broad)** |
| --- | --- | --- |
| Records sampled | 300 | 300 |
| Included after arbitration | 79 | 55 |
| Precision (95% CI) | 26.3% [21.7%, 31.6%] | 18.3% [14.4%, 23.1%] |
| Worst-case scenario | 31.7% | 21.7% |
| All-disagreements-rejected scenario | 25.3% | 16.7% |
| Decision | SELECTED (point estimate 8.0 pp higher; robust across scenarios) | Rejected |

# 6. Eligibility codes

Inclusion required ALL of: (1) publication years 2005–2025; (2) journal article or review; (3) English; (4) nursing-education context (nursing students, nurse educators, or formal nursing programmes); (5) substantive POP-construct focus in one of three evidence forms: (a) the construct measured as an outcome, (b) the construct as the content of an educational intervention, or (c) the construct as a primary qualitative theme.

**Table S6. Eligibility codes**

| **Code** | **Exclusion reason** | **Definition** |
| --- | --- | --- |
| E1 | Population/context mismatch | Not a nursing-education context (e.g., registered nurses in practice, workplace outcomes, clinical nursing practice) |
| E2 | Insufficient construct evidence | No POP construct as measured outcome, intervention content, or primary qualitative theme (e.g., construct named only as a predictor, background citation, or generic deficit framing) |
| E3 | Mixed population not separable | Mixed health-professions or mixed student/practitioner samples in which nursing participants cannot be separated |
| E4 | Document type | Not a journal article or review (editorials, proceedings, book chapters, etc.) |
| E5 | Language | Non-English full text |

# 7. Batch-level double-blind screening statistics

After the 500-record pilot batch (125 disagreements resolved; six principal-investigator referrals; codebook finalized at version 3), 13 full-scale batches were screened independently and in double blind. Every disagreement was adjudicated record by record against the codebook; residual conflicts were decided by the principal investigator. κ varied across batches (0.11–0.63) because the safety-net screener's inclusion-side false-positive rate rose across batches (40%→82%) as the corpus progressed into constructs with lower base rates—an asymmetry known to depress κ without affecting final adjudicated decisions. Observed agreement (Po) ranged 0.76–0.84. In total, 12,361 records underwent paired title/abstract screening across the 14 batches (Table S7).

**Table S7. Batch-level double-blind screening statistics**

| **Batch** | **Records** | **Both include** | **Overruled (safety-net only)** | **Rescued (reviewer only)** | **Po** | **Cohen's κ** |
| --- | --- | --- | --- | --- | --- | --- |
| Pilot | 500 | — | — | — | 0.75 (125 disagreements) | — |
| 2 | 500 | 32 | 113 | 7 | 0.758 | — |
| 3 | 500 | 100 | 70 | 29 | 0.802 | — |
| 4 | 1,000 | — | 119 | — | — | 0.113 |
| 5 | 1,000 | 16 | 114 | 9 | — | 0.172 |
| 6 | 1,000 | 66 | 58 | 10 | — | 0.625 |
| 7 | 1,000 | 239 | 98 | 70 | — | 0.616 |
| 8 | 1,000 | 235 | — | 51 | — | 0.629 |
| 9 | 1,000 | 230 | 145 | 66 | — | 0.528 |
| 10 | 1,000 | 249 | 126 | 65 | 0.808 | 0.578 |
| 11 | 1,000 | 215 | — | 59 | — | 0.537 |
| 12 | 1,000 | 178 | 119 | 36 | 0.843 | 0.594 |
| 13 | 1,000 | 82 | 156 | 29 | 0.815 | 0.375 |
| 14 | 861 | 41 | 191 | 16 | 0.793 | 0.212 |
| Total | 12,361 | — | — | — | — | — |

# 8. Author-name disambiguation (four-step deterministic procedure)

(1) Full-name parsing with diacritic folding and surname anchoring—identical full names are treated as the same person. (2) Hard merge of name variants sharing an ORCID (57 groups). (3) Affiliation-anchored absorption: variants with the same surname, compatible given-name initials, and either overlapping affiliations or co-author bridges were merged (144 variant pairs in 137 groups, each with documented evidence). (4) Hard block: records carrying two different ORCIDs were never merged. Residual collision risk is bounded at ≤21 name keys (0.27% of 7,728 disambiguated authors), all in the long tail; no top-20 author is affected.

**Table S8. Top 10** **most productive authors after disambiguation.**

| **Rank** | **Author** | **Publications** | **Total citations (in-collection)** | **Affiliation** | **ORCID** |
| --- | --- | --- | --- | --- | --- |
| 1 | Tracy Levett-Jones | 14 | 1,182 | University of Newcastle, Australia | 0000-0003-4279-8957 |
| 2 | Mohamed Hussein Ramadan Atta | 12 | 117 | Prince Sattam Bin Abdulaziz University, Saudi Arabia | — |
| 3 | Daniel Joseph E. Berdida | 10 | 303 | University of Santo Tomas, Philippines | 0000-0002-5001-6946 |
| 4 | Jonas Preposi Cruz | 10 | 230 | Shaqra University, Saudi Arabia | — |
| 5 | Cayetano Fernandez-Sola | 10 | 154 | Universidad de Almería, Spain | 0000-0003-1721-0947 |
| 6 | Jose Manuel Hernandez-Padilla | 10 | 154 | Middlesex University, UK | 0000-0002-5032-9440 |
| 7 | Chaoran Chen | 10 | 152 | Henan University, China | — |
| 8 | Leodoro J. Labrague | 9 | 266 | Sultan Qaboos University, Oman | 0000-0003-0315-4438 |
| 9 | Huan Liu | 9 | 194 | Central South University, China | — |
| 10 | Lorna Moxham | 9 | 171 | University of Wollongong, Australia | 0000-0002-4127-6383 |

# 9. Cited sources, top-cited publications, and author impact

Table S9 lists the ten most frequently cited sources in the reference base of the included publications, led by Nurse Education Today (7,050 citations), Nurse Education in Practice (2,433), and Journal of Advanced Nursing (2,123), indicating the journals on which the knowledge base of this field predominantly draws. Table S10 presents the ten most cited publications in the field, each annotated with the POP construct it anchors and the form of evidence; the list is led by simulation and virtual-simulation learning studies and resilience research, identifying the intellectual landmarks of the field.

**Table S9. Top 10 cited sources in the reference base.**

| **Rank** | **Source** | **Citations** |
| --- | --- | --- |
| 1 | NURS EDUC TODAY | 7,050 |
| 2 | NURSE EDUC PRACT | 2,433 |
| 3 | J ADV NURS | 2,123 |
| 4 | J NURS EDUC | 1,775 |
| 5 | BMC MED EDUC | 1,014 |
| 6 | BMC NURS | 969 |
| 7 | J PROF NURS | 954 |
| 8 | INT J ENV RES PUB HE | 934 |
| 9 | INT J NURS STUD | 925 |
| 10 | CLIN SIMUL NURS | 911 |

**Table S10. The top 10 cited publications about POP in nurse education. The final column documents the POP construct anchor of each publication, verified at full-text level (evidence forms: (a) construct measured as an outcome; (b) construct as intervention content; (c) construct as a primary qualitative theme).**

| **Authors, Year, Journal** | **Title** | **DOI** | **Document type** | **Total citations (WoS)** | **Citations per year** | **POP construct anchor (evidence form)** |
| --- | --- | --- | --- | --- | --- | --- |
| Cant, RP; Cooper, SJ, 2017, Nurse Education Today | Use of simulation-based learning in undergraduate nurse education: An umbrella systematic review | 10.1016/j.nedt.2016.11.015 | Review | 392 | 43.6 | Self-efficacy (measured outcome effect size); self-confidence \| (a) |
| Padilha, JM; Machado, PP; Ribeiro, A; Ramos, J; Costa, P, 2019, Journal of Medical Internet Research | Clinical Virtual Simulation in Nursing Education: Randomized Controlled Trial | 10.2196/11529 | Article | 350 | 50 | Self-efficacy + satisfaction with learning (co-primary outcomes) \| (a) |
| Li, ZS; Hasson, F, 2020, Nurse Education Today | Resilience, stress, and psychological well-being in nursing students: A systematic review | 10.1016/j.nedt.2020.104440 | Review | 282 | 47 | Resilience + psychological well-being (co-focus in title/aim) \| (a) |
| Song, Y; Lindquist, R, 2015, Nurse Education Today | Effects of mindfulness-based stress reduction on depression, anxiety, stress and mindfulness in Korean nursing students | 10.1016/j.nedt.2014.06.010 | Article | 277 | 25.2 | Mindfulness (MBSR intervention itself) \| (b) |
| Levett-Jones, T; Lathlean, J; Higgins, I; McMillan, M, 2009, Journal of Advanced Nursing | Staff-student relationships and their impact on nursing students' belongingness and learning | 10.1111/j.1365-2648.2008.04865.x | Article | 265 | 15.6 | Sense of belonging (qualitative main theme) \| (c) |
| Por, J; Barriball, L; Fitzpatrick, J; Roberts, J, 2011, Nurse Education Today | Emotional intelligence: Its relationship to stress, coping, well-being and professional performance in nursing students | 10.1016/j.nedt.2010.12.023 | Article | 230 | 15.3 | Emotional intelligence (scale-measured) + well-being \| (a) |
| van der Riet, P; Levett-Jones, T; Aquino-Russell, C, 2018, Nurse Education Today | The effectiveness of mindfulness meditation for nurses and nursing students: An integrated literature review | 10.1016/j.nedt.2018.03.018 | Review | 212 | 26.5 | Mindfulness/meditation (intervention itself) \| (b) |
| Chang, CY; Hwang, GJ; Gau, ML, 2022, British Journal of Educational Technology | Promoting students' learning achievement and self-efficacy: A mobile chatbot approach for nursing training | 10.1111/bjet.13158 | Article | 204 | 51 | Self-efficacy (title-level measured outcome) \| (a) |
| Edwards, D; Burnard, P; Bennett, K; Hebden, U, 2010, Nurse Education Today | A longitudinal study of stress and self-esteem in student nurses | 10.1016/j.nedt.2009.06.008 | Article | 183 | 11.4 | Self-esteem (co-focus in title; CFSEI-measured) \| (a) + defect-framework rule (i) |
| Montes-Berges, B; Augusto, JM, 2007, Journal of Psychiatric and Mental Health Nursing | Exploring the relationship between perceived emotional intelligence, coping, social support and mental health in nursing students | 10.1111/j.1365-2850.2007.01059.x | Article | 180 | 9.5 | Emotional intelligence (TMMS-measured; co-focus in title) \| (a) + defect-framework rule (i) |

# Supplementary figures

**Figure S2. Country collaboration network.**

**Figure S3. Keyword co-occurrence network (50 nodes, 353 edges; Louvain clustering).**

**Figure S4. Thematic evolution of keyword communities across the three pre-specified periods (2005–2013, 2014–2020, 2021–2025).**

**Figure S5. Thematic map (motor, basic, niche, and emerging/declining themes).**
